# Effect of lifestyle interventions of pregnant women on their dietary habits, lifestyle behaviors, and weight gain: a randomized controlled trial

**DOI:** 10.1186/s41043-016-0044-2

**Published:** 2016-02-24

**Authors:** Özlem Aşcı, Gülay Rathfisch

**Affiliations:** 1 Nursing Department of Health Sciences Faculty, Artvin Çoruh University, Çayağzı Mah. Liflevha Sok., 0800 Artvin, Turkey; 2 Nursing Faculty of Obstetrics Nursing Department, Florence Nightingale School of Nursing, Istanbul University, Abidei Hurriyet cad., 34381 Istanbul, Turkey

**Keywords:** Dietary and lifestyle interventions, Exercise, Gestational weight gain, Pregnancy, Weight retention, Health-promoting lifestyle behavior

## Abstract

**Background:**

Although it is known that lifestyle behaviors of pregnant women are closely related to maternal and fetal health, number of data concerning efficacy of intervention on lifestyle during pregnancy is limited. The purpose of this study is to determine the effect of lifestyle interventions on improving dietary habits and lifestyle behaviors, ensuring gestational weight gain (GWG) within recommended levels and limiting postpartum weight retention (PWR).

**Methods:**

The study was conducted as a randomized controlled trial in a family health center located in Istanbul, Turkey, between June 2011 and July 2012. The primary outcomes were GWG, and the proportion of pregnant women whose GWG was within the Institute of Medicine (IOM) guidelines. One hundred two pregnant women with gestation ≤12 weeks, age ≥18 years, gravidity ≤2, and who did not intend to lose weight in prepregnancy period were randomly included in this study as intervention (*n* = 51) and control (*n* = 51) groups. The study was completed with 45 women for each group. The control group received routine antenatal care. The intervention group was received an individualized lifestyle intervention focusing on healthy lifestyle, diet, exercise, and weight monitoring as four sessions at 12–15, 16–18, 20–24, and 37 weeks gestation. Lifestyle behaviors were evaluated with Health-Promoting Lifestyle Profile-II. Dietary habits were assessed by 3-day dietary recalls, and weight was followed from pregnancy until 6 weeks postpartum.

**Results:**

The lifestyle interventions had a significant effect on improving lifestyle behaviors, protein intake, percentage of energy from protein, calcium, magnesium, iron, zinc, and vegetable intakes when adjusted for confounders (*p* < 0.05). The proportion of women who were within the IOM recommendations was higher in the intervention group (51.1 %) than in the control group (28.9 %) The odds ratio for GWG within IOM was statistically significant between the groups (OR = 0.59, 95 % CI, 0.45–0.72). There were no difference between groups in terms of the other dietary intakes, total GWG, and PWR (*p* > 0.05).

**Conclusions:**

Lifestyle intervention improves the lifestyle behaviors during pregnancy and increases the appropriate GWG for prepregnancy body mass index (BMI), but it has a limited effect in terms of improving dietary habits and has no effect on PWR.

## Background

Obesity is a common disease with high mortality and morbidity rates [1]. According to the Turkish Nutrition and Health Survey (2010), 70.7 % of Turkish women are overweight and/or obese [2]. Studies indicate that obesity is more common among women than men [1, 2] and that excessive gestational weight gain (GWG) and postpartum weight retention (PWR) contribute to overweight/obesity among women in the long term [3, 4]. Excessive GWG is also related to pregnancy complications, infant macrosomia, the increase in cesarean section rates, and PWR [3, 4]. The Institute of Medicine (IOM) in the USA suggests that prepregnancy body mass index (BMI) is a base for determining the optimal GWG range. Appropriate GWG for prepregnancy BMI is important for positive fetal and maternal pregnancy results [5]. Since there are no GWG guidelines based on prepregnancy BMI in Turkey, generally IOM guidelines are used. Regardless of their BMI, it is recommended that all women to receive consultancy in terms of healthy lifestyle, nutrition, and physical activity during pregnancy [6]. Therefore, it is important to determine effective interventions on developing a healthier lifestyle for pregnant women in order to improve maternal and fetal health and bring current or possible obesity and related health problems under control [7–9].

Interventions focusing on diet, physical activity, and weight gain and aiming to develop a healthier lifestyle are known to be effective in preventing and controlling obesity [10]. However, the effect of these interventions (dietary intervention with or without increased physical activity) in controlling GWG and decreasing PWR among pregnant women is not clear [8, 11–20]. In addition, it is indefinite how much interventions applied in the studies improve healthy lifestyle adaptation, dietary and physical activity habits, and the consultancies given in which periods by whom were more effective. Besides, some studies include only obese and overweight women or focus on reducing excessive weight gain rather than the appropriate GWG for prepregnancy BMI [12, 13, 15–17, 19]. However, there are results indicating that women who are underweight in prepregnancy periods may become overweight during pregnancy and appropriate GWG is important for all women [5, 9]. Consequently, due to the differences in the study groups, insufficiencies in the quality of studies and a limited number of studies, the success of lifestyle interventions on GWG is not clearly understood and it is required to conduct the related studies [21].

The purpose of this study is to determine the effect of lifestyle intervention, offered within the scope of antenatal care, on adapting to a healthy lifestyle, developing dietary habits, ensuring GWG to be within the levels recommended by 2009 IOM guidelines, and lessening the PWR.

## Methods

### Design and participants

This study was conducted with the randomized controlled trial. Permissions were obtained from Istanbul University Cerrahpaşa Medical Faculty Ethics committee and Provincial Directorate of Health of Istanbul for this study. The study was conducted between June 2011 and July 2012 in Istanbul in a family health center providing services for a population of approximately 21,000 people. In Turkey, doctors generally work in cooperation with midwives and nurses in family health centers. In these centers, efficient, easily accessible, and free mother-child health services are provided. The center where the study was conducted is located in a region receiving internal migration, mostly consisting of families with middle income levels and having intense use of health services.

In this study, GWG and the proportion of pregnant women whose GWG was within the Institute of Medicine (IOM) guidelines were the primary outcomes. The lifestyle behaviors, dietary habits and PWR were the secondary outcomes.

**Fig. 1:**
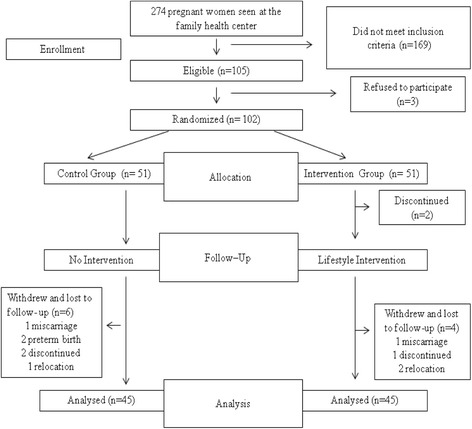
Flow chart of the study

In calculation of sample size of the study, a prior power analysis was conducted by using G Power 3.1.9.2 program. According to this analysis, the size of sample calculated with a power level of 80 % for Student’s *t* test with a middle effect size (*d* = 0.5, *α* = 0.05) was determined to be 102 people. One hundred two women out of 274 pregnant women receiving services from the family health center agreed to participate in the study. Pregnant women aged over 18, who had no health problem, did not intend to lose weight in prepregnancy period, got pregnant in natural ways for two times at most, and were pregnant for a period of 3 months or less, were included in the study. The women were divided into randomized groups by a staff who was not involved in this study, by drawing lots (control *n* = 51, intervention = 51). Participants were blind about which group they were involved in and the evaluated study outcomes. Groups were interviewed at different times and in different rooms in the center in order to control the interaction between groups. Six people from intervention and control groups were excluded from the study due to reasons such as not coming to regular follow-ups and pregnancy complications. The study was completed with 90 people (Fig. 1). Each participant was informed that their information would be kept in confident, and they had a chance to reject participating in the study and withdraw from the study at any time.

### Procedures of data collection

Groups were followed up in the period starting before the 12th week of pregnancy and continuing until the postpartum 6th week. Data of the study were collected by researchers through one-to-one interviews made with participants. After women were divided into the groups, questionnaires involving socio-demographic characteristics and descriptive features concerning the current pregnancy and being formed by researchers were applied in this study. Heights and weights of women were measured. Their prepregnancy weights were recorded according to their statements. Prepregnancy BMI values were calculated according to the mean of these measurements and statements. Women with a prepregnancy BMI value of <18.5 kg/m^2^ were considered underweight, women with a value between 18.5 and 24.9 kg/m^2^ were considered normal, women with a value between 25 and 29.9 kg/m^2^ were considered overweight, and women with a value of 30 and above were considered obese. Afterwards, in order to evaluate all women with the data within the same pregnancy week, dietary habits and lifestyle behaviors of women were assessed in the gestational 12th (pretest) and 37th (posttest) weeks. In the postpartum 6th week, weights were measured in order to determine the PWR and obstetrical and neonatal outcomes (complications, birth method, hospitalization period, and newborn weight and height) were measured by using the questionnaires formed by researchers.

### Measurements

Calibrated mechanic weighing machines and height gauges were used in order to measure the weight and height of women. These measurements were repeated twice, and the mean was taken. Weights of women were measured with light clothes and no shoes. IOM guidelines suggest that women who were underweight before pregnancy should gain 12.5–18 kg, women who had normal weight should gain 11.5–16 kg, women who were overweight should gain 7–11.5 kg, and women who were obese should gain 5–9.1 kg [5]. In this study, women were evaluated based on whether GWG is gained within these levels recommended by IOM guidelines or not.

Nutritional data were obtained by recording all foods and beverages consumed by women for 3 days (two weekdays and one weekend day). In order to teach women how to record the foods they consume and understand portion amounts, the forms were filled by researchers together with the women on the first day by asking them what they consumed in their meals in the previous day. Women filled their dietary recall in the forms at their home for the remaining days and brought them back for the next follow-up.

Healthy lifestyle behaviors were measured by using the Profile-II consisting of six subscales developed by Walker et al. and revised in 1996 [22, 23]. These six subscales are nutrition, physical activity, spiritual growth, interpersonal relationship, health responsibility, and stress management. Nutrition subscale aims to determine “changes made by individuals while choosing and arranging their meals and their food choices,” physical activity subscale, and “at what level exercises which constitute an essential part of a healthy life are done.” While the lowest score of the scale is 52, the highest score is 208. The lowest score for nutrition subscale is 9.0 and the highest score is 36.0; the lowest score for physical activity subscale is 8.0 and the highest score is 32.0. Higher scores indicate a healthier lifestyle [23, 24]. In this study, the scale which was developed by Walker et al., [23] and whose Turkish validity and reliability was conducted by Bahar et al., [24] was used. General alpha value of the scale was found as 0.92 by Bahar et al [24]. The alpha value of the scale was determined as 0.87 in this study.

### Standard care

Women are generally followed up by at least four times by midwives or nurses in standard care. In every follow-up, weights of women are measured; however, they are not informed on what the GWG range appropriate for their BMI is and their personal weight changes. Consultancies mostly consist of subjects such as pregnancy complaints, scope of antenatal care, tests to be performed, birth, postpartum period, and circumstances that might pose danger during pregnancy. Interventions are not performed to help adapt to a healthier lifestyle. There is no standard training and consultancy.

### Lifestyle intervention

The intervention was applied exactly by the first author of this study. The intervention was derived from previous studies [12, 17] and recommendations in national guidelines [6, 25, 26]. Four meetings were held with women regarding healthy lifestyle, nutrition, exercise, and weight follow-up. At the beginning of interviews, a card indicating personal height, weight, and appropriate GWG range for BMI was prepared for every woman. This card was given to the woman for reminder. She was asked to bring along this card. Weights were measured in every meeting. GWG was recorded on this card. At every meeting, objectives of nutrition and physical activity for optimal GWG were specified until the next meeting. Women reaching their objectives were praised and encouraged. Nutrition and physical activity levels of women who could not reach their objectives were reviewed with women, and a more intensive consultancy (repetition of basic nutrition and physical activity recommendations, reviewing individual objectives, and supportive phone consultancy) was provided. Pender’s health promotion model was used in order to allow women express their experiences and opinions through open-ended questions—e.g., what problems (barriers) you may have in order to eat healthier foods (more vegetables, more fruits, lower fat foods, and healthy grains)?—regarding nutrition and physical activity [27]. Therefore, counseling and behavioral skill-building interventions were personalized according to the barriers for the individuals to displaying the behavior and their self-efficacies in terms of performing the behavior. Interviews were conducted by asking open-ended questions, using reflected listening and affirmation statements [28]. In interviews made in weeks 12–15, 16–18, and 20–24, a 15-min health training prepared in the computer was carried out and then brochures were delivered. Each of these interviews lasted for about 1 h. In gestational weeks 12–15, the women were interviewed regarding the importance of healthy life and health practices. Optimal GWG target, which is appropriate to prepregnancy BMI, was determined. Women were informed about the importance of gaining weight within the recommended range and maintaining a healthy life [29]. In gestational weeks 16–18, interviews were held concerning physical activity and exercises. Low-level aerobic exercises recommended for pregnancy were shown and performed. Women were recommended to do mild-moderate safe exercise types, which increase the heart rate to maximum 140 beats/min while being easily able to talk, for 30 min every other day (elliptical trainer, swimming, plates, yoga, golf adapted for pregnancy, and mild level aerobic exercises), and maintain a more active lifestyle (taking walks every day, going to work by walking, using stairs instead of elevators, participating in sportive activities in their leisure times).

In gestational weeks 20–24, interviews regarding nutrition were held. Women were informed about the basic nutrition principles (eating small but frequent meals for at least 5 times a day, having breakfast every day, portions and amounts required to be consumed from all food groups, lessening consumption of sweet foods to once a day or less, increasing fibrin intake from bread, and decreasing fat in diet). Recommendations for consuming more healthy foods (e.g., fruit) instead of foods containing intensive energy (e.g., fast food and sweets) without any energy limitation according to the personal dietary habits were given. In week 37, only weights were followed up and the women’s status of reaching targets was reviewed. The intervention was ended with this interview. In the postpartum 6th week, weights and obstetrical and neonatal outcomes were measured.

### Statistical analysis

Statistical analysis of data was conducted by using Statistical Package for the Social Sciences (SPSS) Version 16.0. Data obtained from food consumption forms were analyzed by using Nutrition Information System (BeBIS) program (ver. 7.1.) which is a computer program adapted to Turkish nutrition culture and used for determining nutritional status of individuals. *p* < 0.05 was used as the level of statistical significance. Kolmogrov Smirnov and Levene tests were used for normal distribution and homogeneity evaluation of the data. All continuous variables had normal distribution and equal variances. Differences between groups in terms of characteristics and obstetrical and neonatal outcomes were analyzed by using Student’s *t* test for continuous variables and chi-square test for categorical variables. When cell counts were <5, Fisher’s exact test was used. Characteristics (age, education year, prepregnancy BMI, gestational age at recruitment, income level, intended pregnancy status, and gravidity) were included when necessary as confounding factors in the multivariable analyses. Logistic regression analysis was used to examine the effects of intervention and confounding factors on the proportion of women who were within the IOM recommended level. The groups of women with weight gain below or exceed the recommendation were combined in this analysis. Pretest and posttest differences between groups in terms of healthy lifestyle behaviors and dietary intakes were analyzed by using Student’s *t* test. Repeated measures analysis of covariance (ANCOVA) was used to evaluate group differences adjusted for pretest values of healthy lifestyle behaviors and dietary intakes, prepregnancy age, BMI, education year, and gravidity. Bonferroni correction was used for these variables.

## Results

No significant difference was observed between the groups in terms of age, education year, working status, income level, status of intended pregnancy, prepregnancy BMI, gravidity, and the time of inclusion according to the data obtained in the beginning of the study (Table 1).

**Table 1:** Characteristics of pregnant women in control and intervention groups

Variables	Control	Intervention	*p* value^a^
Age (years)	24.28 ± 4.15	24.31 ± 4.22	0.989
Education mean (years)	6.6 ± 2.8	7.6 ± 3.2	0.213
Prepregnancy BMI (kg/m^2^)	22.82 ± 3.93	23.86 ± 4.10	0.285
Gestational age at recruitment (weeks)	6.64 ± 2.66	7.95 ± 2.85	0.160
Working status [*n* (%)]	2 (4.4)	7 (15.6)	0.079
Income [*n* (%)]			
ᅟLow	11 (24.4)	15 (33.3)	0.220
ᅟMiddle	34 (75.6)	30 (66.7)	
ᅟHigh	–	–	
Intended pregnancy [*n* (%)]	40 (89.9)	38 (84.4)	0.589
Gravidity [*n* (%)]			
ᅟ1	25 (55.6)	29 (64.4)	0.486
ᅟ2	20 (44.4)	16 (35.6)	

Continuous values are expressed as mean ± standard deviation. Control, *n* = 51; intervention, *n* = 51

*BMI* body mass index

^a^There were no significant differences between groups, *p* > 0.05

According to the measurements obtained in the 12th week of pregnancy (pretest), there was no significant difference between HPLP II total and subscale mean scores of groups (*p* > 0.05). In the 37th week of pregnancy (posttest), HPLP II total (*p* < 0.001), nutrition (*p* < 0.05), and physical activity (*p* < 0.001) subscale mean scores were significantly higher in the intervention group compared to the control group. When adjusted for baseline intake of the outcome variable (pretest), prepregnancy age, BMI, education year, and gravidity, there was a significant difference between HPLP II total, nutrition, and physical activity posttest scores (*p* < 0.05). The lifestyle intervention had a significant effect on health-promoting behavior, nutrition, and physical activity, when adjusted for baseline intake of the outcome variable (pretest), prepregnancy age, BMI, education year, and gravidity (Table 2). According to these findings, it was concluded that the lifestyle intervention in the study was efficient in terms of adapting pregnant women to a healthier lifestyle and improved the physical activity and nutritional behaviors of women.

**Table 2:** Lifestyle behaviors of groups and adjusted group differences (95 % CI) of posttest

Variables	Pre	Post	Adjusted mean difference to controls^a^	*p* value^a^
Health-promoting lifestyle behavior				
Control	122.75 ± 17.66	125.11 ± 16.86		
Intervention	124.26 ± 16.96	138.22 ± 16.86	+11.02 (4.52–17.51)	0.001
Nutrition				
Control	21.62 ± 3.53	22.48 ± 4.26		0.023
Intervention	21.63 ± 4.68	23.33 ± 3.29	+1.62 (0.23–3.02)	
Physical activity				
Control	12.06 ± 4.24	14.04 ± 4.21		
Intervention	12.60 ± 3.42	17.91 ± 4.30	+3.12 (1.51–4.74)	0.0002

*CI* confidence interval. Missing for each groups, *n* = 6. Control, *n* = 45; intervention, *n* = 45

^a^ANCOVA, mean group differences adjusted for baseline intake of the outcome variable (pretest), prepregnancy age, BMI, education year, and gravidity, *p* < 0.05

According to pretest measurements, no significant differences were observed between the nutritional data of groups in the 12th week of pregnancy (*p* > 0.05). Examining pretest and posttest nutritional data measurements of groups obtained with a 6-month interval, it was determined that in the 37th week of pregnancy, protein intake (*p* = 0.013), percentage of energy from protein (*p* = 0.032), dietary fiber (*p* = 0.044), calcium (*p* = 0.032), magnesium (*p* = 0.024), iron (*p* = 0.027), zinc (*p* = 0.003), fruit (*p* = 0.032), and vegetable intakes (*p* = 0.007) were significantly higher in the intervention group compared to the control group. There was no significant difference between the other dietary intakes of control and intervention groups (*p* > 0.05). After mean group differences were adjusted for pretest, prepregnancy age, BMI, education year, and gravidity (Table 3), the lifestyle intervention had only a significant effect on improving protein intake, percentage of energy from protein, calcium, magnesium, iron, zinc, and vegetable intakes (*p* < 0.05). In line with these findings, it was concluded that the lifestyle intervention had a limited effect on improving the dietary habits.

**Table 3:** Dietary intakes of groups and adjusted group differences (95 % CI) of posttest

Groups	Control		Intervention		Adjusted mean difference to controls^a^	
Variables	Pre	Post	Pre	Post		*P* value^a^
Energy intake (kkal/day)	1867 ± 587	1888 ± 581	1731 ± 638	2087 ± 664	+222 (−54–499)	0.113
Protein intake (g)	58.6 ± 5.8	58.9 ± 5.8	55.6 ± 2.3	73.6 ± 3.3	+15.6 (4.1–27.1)	0.008^b^
(% of energy)	13 ± 0	12 ± 8	13 ± 2	14 ± 4	+1.9 (0.2–3.7)	0.025^b^
Carbohydrate intake (g)	242.7 ± 96.7	237.2 ± 94.2	219.6 ± 89.4	255.7 ± 107.6	+23.5 (−21.6–68.6)	0.304
(% of energy)	34 ± 7	36 ± 6	35 ± 6	35 ± 7	−0.5 (−5.3–4.1)	0.806
Fat intake (g)	71.2 ± 23.8	75.9 ± 21.3	67.7 ± 28.0	82.9 ± 25.0	+7.8 (−2.3–18.1)	0.130
(% of energy)	52 ± 7	50 ± 7	51 ± 7	49 ± 8	−1.7 (−6.9–3.5)	0.512
Dietary fiber (g/day)	21.5 ± 7.8	22.3 ± 8.2	21.7 ± 7.5	25.7 ± 8.3	+3.2 (−0.4–6.9)	0.084
Calcium intake (mg/day)	652.8 ± 20.6	682.2 ± 282.9	688.3 ± 32.4	813.7 ± 346.7	+152.6 (14.7–290.5)	0.030^b^
Magnesium intake (mg/day)	235.2 ± 63.0	246.8 ± 78.4	235.7 ± 87.1	289.5 ± 96.3	+43.1 (4.1–82.0)	0.031^b^
Iron (mg)	9.8 ± 3.0	10.1 ± 3.4	9.8 ± 3.3	11.8 ± 3.6	+1.7 (0.2–3.3)	0.025^b^
Zinc (mg)	9.1 ± 2.8	8.8 ± 2.5	8.8 ± 3.3	10.7 ± 3.4	+2.0 (0.7–3.3)	0.003^b^
Folate (μcg)	282.7 ± 88.5	284.2 ± 91.3	265.8 ± 92.0	296.0 ± 114.7	+12.0 (−32.7–58.8)	0.573
Fruit intake (pieces/day)	1.6 ± 1.1	1.5 ± 1.0	1.7 ± 1.0	2.1 ± 1.3	+0.4 (−0.5–1.04)	0.078
Vegetable intake (g/day)	213 ± 108	204 ± 129	176 ± 102	283 ± 132	+73. 6 (12.5–134.7 )	0.019^b^

*CI* confidence interval. Missing for each groups, *n* = 6. Control, *n* = 45; intervention, *n* = 45

^a^ANCOVA, mean group differences adjusted for baseline intake of the outcome variable (pretest), prepregnancy age, BMI, education year, and gravidity

^b^There were significant differences between groups, *p* < 0.05

According to the measurement performed in the intervention and control groups in the 37th week of pregnancy, the total mean GWG values were respectively 12.45 ± 5.04 and 12.29 ± 4.80 kg (*p* > 0.05). Within the range recommended by 2009 IOM guidelines, the GWG values were significantly higher in the intervention group than in the control group (51.1 versus 28.9 %, chi-square 4.6296, *p* < 0.05). According to the results of logistic regression analysis, the odds ratio (OR) for GWG within IOM showed a statistically significant difference between the groups (OR = 0.59, 95 % CI, 0.45–0.72). When adjusted for confounders, the odds ratio for GWG within IOM was 0.379 (95 % CI, 0.141–1.02) with borderline *p* value significance (Table 4). In line with these findings, it was concluded that the lifestyle intervention was efficient in terms of ensuring the GWG within the range recommended by 2009 IOM guidelines.

**Table 4:** GWG, PWR, and obstetrical and neonatal outcomes of groups and OR (95 % CI)

Variable	Control	Intervention	*p* value
GWG (kg)	12.29 ± 4.80	12.45 ± 5.04	0.87
GWG within IOM [*n* (%)]	13 (28.9)	23 (51.1)	0.03^a^
OR for GWG within IOM (95 % CI)	1.00 (ref.)	0.593 (0.459–0.726)	0.008^b^
Adjusted OR for GWG within IOM (95 % CI)	1.00 (ref.)	0.379 (0.141–1.021)	0.05^c^
PWR (kg)	5.95 ± 4.79	5.19 ± 4.71	0.44
Gestational age (w)	39.33 ± 1.34	39.33 ± 1.34	0.97
Cesarean section [*n* (%)]	15 (31.1)	17 (37.8)	0.50
Hospitalization time (h)	30.26 ± 20.91	28.93 ± 18.8	0.75
Birth weight (kg)	3.298 ± 4.23	3.268 ± 3.80	0.76
Infant length (cm)	50.40 ± 1.90	50.04 ± 1.78	0.29

Continuous values are expressed as mean ± standard deviation. Missing for each groups, *n* = 6. Control, *n* = 45; intervention, *n* = 45

*CI* confidence interval, *GWG* gestational weight gain, *IOM* Institute of Medicine (2009), *PWR* postpartum weight retention, *OR* odds ratio

^a^ *X* ^2^ test

^b^Logistic regression model

^c^Logistic regression model, adjusted for age, education year, prepregnancy BMI, gestational age at recruitment, income level, intended pregnancy status, and gravidity

According to the measurement performed in the sixth postpartum week, participants expressed that they did not experience any health problem during pregnancy and in the postpartum period. No difference was observed between groups in terms of the obstetrical and neonatal outcomes and PWR in the study (*p* > 0.05) (Table 4).

## Discussion

In this randomized controlled study, an intervention that aims at controlling the GWG by developing a healthier lifestyle and is offered through one-to-one interviews was designed. Nutrition and exercise recommendations in the national guidelines were based on previous studies in order to design the intervention by considering the fact that they can be easily applied with a low cost in clinics by nurses and midwives within the scope of antenatal care. The intervention focused on healthy lifestyle, nutrition, physical activity, and weight follow-up. Personal weight change was given to women in writing after having measured their weights for four times during pregnancy. Motivational interview, HPM-based consultancy and goal-setting strategies were used altogether.

This intervention had a significant effect on GWG within the range recommended by IOM guidelines (OR = 0.59, 95 % CI 0.45–0.72). It significantly increased the GWG prevalence within the range recommended by IOM guidelines (28.9 versus 51.1 %). This finding is consistent with the studies revealing that lifestyle interventions are successful in terms of ensuring that the GWG is within the range recommended by IOM guidelines mostly among women with normal weights [15, 20]. In this study, no difference was observed between groups in terms of the total mean GWG. Contrary to our study, Asbee et al. [17] reported that lifestyle interventions decreased the total GWG but were insufficient in terms of providing weight gain within the range recommended by IOM guidelines. However, in line with our study, the results of the recent studies which analyze the effect of lifestyle interventions on GWG have revealed that lifestyle interventions are ineffective or have a low effect on the total GWG [14, 30].

Food intakes are critical for the management of gestational weight gain [16]. Changes in GWG partially depended on the intensity of dietary interventions [30]. In this study, according to the measurements performed with approximately 6-month interval, lifestyle intervention has a limited effect on developing the dietary habits. In several studies [12, 17, 31], the effect of lifestyle interventions on limited dietary outcomes (only energy intake or fat intake, bread containing high fibrin, vegetable and fruit intake) was examined. Polley et al. [17] found no effect of a stepped care behavioral intervention on changing the fat intake from 13 high-fat foods among pregnant women in the measurements made with 10-week intervals. Kinnunen et al. [12] determined that individual counseling on diet and physical activity during pregnancy helped pregnant women to maintain the proportion of high-fiber bread and to increase vegetable, fruit, and fiber intakes. Rauh et al. [31] found that the lifestyle intervention group had a lower energy intake than the control group when comparing the differences between groups in terms of changes from baseline to the 36–38th week interval of gestation. Only two studies which aim to control the GWG through lifestyle interventions including only pregnancy period and examine the effect of the interventions on the extended dietary outcomes (such as daily energy, macro and micro food components intake, and vegetable and fruit consumption) as in this study were found [13, 16]. The first of them is the study conducted by Guelinckx et al. [13], only with the obese and overweight women. In their study, when both lifestyle interventions including the active training or dietary consultancy with brochure given by dietitians in accordance with the national guidelines regarding healthier nutrition were compared with the first trimester, it was observed that they were efficient in developing diet habits in the last trimester but inefficient on physical activity and GWG. The second one is the study of Hui et al. [16] where they used lifestyle interventions including providing consultancy by dietitian using a computerized dietary interview tool (Food Choice Map). In their study, lifestyle intervention was efficient in improving dietary habits and physical activities and decreasing excessive GWG according to the measurements conducted with an interval of 2 months. In our study, the fact that lifestyle intervention was unsuccessful in improving dietary habits may have affected the power of the intervention to control GWG negatively. This may point out that the nutrition recommendations in national guidelines shall be revised with the aim of improving dietary habits. However, as is seen, there are no sufficient evidences to come to this conclusion. Future studies may focus on evaluating the effect of lifestyle interventions performed during pregnancy on developing dietary habits through extended dietary outcomes and demonstrating the effect of developing dietary habits during pregnancy on GWG and in the long term, on BMI.

Health-promoting lifestyles are “viewed as a multi-dimensional pattern of self-initiated actions and perceptions that serve to maintain or enhance the level of wellness, self-actualization, and fulfillment of the individual” [27]. Health-promoting lifestyle is a very important aspect of health promotion for pregnant women and their offspring [32]. However, few studies have focused on the effects of lifestyle interventions on health promoting lifestyle or related factors (such as self-efficacy, body image, depression, and social support) in pregnancy. Huang et al. [7] reported that diet and physical activity intervention among Taiwanese childbearing women had a significant effect on health-promoting behaviors. Kieffer et al. [33] reported that a community-planned, culturally tailored healthy lifestyle intervention (Healthy MOMs Lifestyle Intervention) led by community health workers can reduce depressive symptoms among pregnant Spanish-speaking Latinas. In this study, the lifestyle intervention was effective in terms of adapting to a healthy lifestyle and especially delivering the habit of physical activity and nutrition behaviors among Turkish pregnant women.

Another study outcome examined in this study is PWR. Phelan et al. [20], reported that behavioral intervention limited with only pregnancy period was not effective in decreasing the PWR in the 6th postpartum month. Althuizen et al. [14] reported that according to the follow-up results of 8th, 26th, and 56th postpartum weeks, dietary and activity consultancy starting with pregnancy and continuing with postpartum phone consultancy was not effective on PWR. In our study, lifestyle intervention which included only the pregnancy period was not efficient in decreasing the weight retention in the 6th postpartum week. In line with present studies, also in this study, the fact that interventions limited with pregnancy were found inefficient in decreasing PWR makes us think that interventions should be continued also in the postpartum period. Similarly, Huang et al. [9] reported that interventions starting with pregnancy period and continuing until the 6th postpartum month were effective in decreasing PWR, which supports this opinion. Additionally, it is important that in line with the previous studies [19, 20, 31], the lifestyle intervention in this study did not increase the incidence of pregnancy and birth complication and did not affect obstetric and neonatal outcomes negatively.

This study is the first study in Turkey where the research protocol was tested and has some limitations. Participation in the study was stopped as soon as the sample size determined by the power analysis was obtained without taking case losses into consideration due to the time constraint for the completion of the study. This situation caused that the study was completed with a smaller sample size than planned. Intervention was applied by the same person in the study (first author of this study who was the nurse officially rendering services in the center on the dates that the study was conducted) within official working hours. From these aspects, the intervention was strong in terms of controlling the contents of consultancy for each participant and “realistic” in terms of applicability by nurses and midwives. However, the fact that the study was conducted in only one center and the sample group was limited to healthy pregnant women who did not intend to lose weight and had less than two pregnancies even if they were selected randomly is not sufficient for generalizing the results of the study.

## Conclusions

This study demonstrated that the lifestyle intervention offered by nurses within the scope of antenatal care was effective in terms of ensuring the optimal GWG and developing a healthy lifestyle. Additionally, it also supported the conclusion that lifestyle interventions that include only pregnancy periods were not sufficient in limiting the PWR. This study may give an opinion to the researchers who are willing to test the efficacy of nurses and applicability of interventions in clinics to control the GWG through lifestyle interventions or prevent excessive GWG. Medical personnel, especially nurses, may use the lifestyle interventions, known not to affect the neonatal and obstetric outcomes negatively, for ensuring the optimal GWG. They can benefit from the contribution of these interventions to the lifestyle that promotes health for improving the health of families and ensuring weight management in the long term.
